# Large Ureterocele Associated With a Duplex Collecting System in an Infant: A Case Report

**DOI:** 10.7759/cureus.114034

**Published:** 2026-08-05

**Authors:** Ajoke I Ogunsakin, Maria Veronica M Ponce

**Affiliations:** 1 Pediatrics, Woodhull Medical Center, Brooklyn, USA

**Keywords:** duplex collecting system, hydronephrosis, infant, pediatrics, ureterocele, urology

## Abstract

Ureteroceles, especially large or ectopic ones in duplex systems, present significant management challenges in neonates due to the risk of urinary tract infections (UTIs) and potential for renal scarring.

We present the case of a neonate diagnosed prenatally with a large ureterocele with associated duplex system and hydronephrosis. Postnatal management followed a conservative initial approach with continuous antibiotic prophylaxis (CAP) using amoxicillin, in alignment with the AAP guidelines. The patient remained asymptomatic while awaiting definitive surgical intervention.

This case highlights the efficacy of standardized urinary tract dilation (UTD) grading and the role of CAP in preventing febrile UTIs while bridging patients toward surgical decompression. However, the efficacy of CAP cannot be generalized from this case report.

## Introduction

Ureterocele is a congenital anomaly that involves cystic dilation of the terminal part of the ureter extending into the bladder, urethra, or both [1]. It is usually associated with a duplex collecting system occurring in the upper moiety (pole). It is found incidentally on prenatal ultrasound; if small may be asymptomatic. The most common complications are bladder outlet obstruction and urinary tract infection (UTI) [2], which can lead to renal scarring and hydronephrosis. We present a case of an infant with a large ureterocele in the setting of a duplicated collecting system leading to significant hydronephrosis to emphasize the clinical presentation, diagnostic evaluation, and management considerations in this rare condition. This case highlights the importance of prenatal ultrasound in the detection, as some patients with this condition may be completely asymptomatic despite having progressive obstruction that may lead to lasting complications like renal disease.

## Case presentation

Our patient is a female neonate born at 39 weeks and 2 days of gestation via spontaneous vaginal delivery. Prenatal ultrasound at 20 weeks revealed a duplicated collecting system in the right kidney and a ureterocele in the bladder. Subsequent prenatal scans showed tortuosity of the right ureter and right hydronephrosis with preserved renal parenchyma. Prenatal labs were all within normal limits. Maternal history was remarkable for anemia and asthma, with no history of diabetes or hypertension. The mother was not on any medications other than prenatal vitamins. There was no family history of renal or urological diseases.

Neonatal course

The pediatric team was called to the delivery due to fetal tachycardia. Her delivery was vaginal and spontaneous, and she cried after birth; APGAR scores were 7 and 8 at 1 and 5 minutes, respectively. However, she showed signs of respiratory distress with subcostal retractions and tachypnea at 10 minutes of life. She received positive end-expiratory pressure (PEEP) and was transferred to the neonatal intensive care unit (NICU) for high-flow nasal cannula support (8 L, FiO₂ 30%). On physical examination, she was well-appearing aside from moderate respiratory distress. No dysmorphic features were noted; the lungs were clear bilaterally, with no murmurs on cardiac examination. Her abdomen was soft and non-tender, with no masses or organomegaly. Normal female external genitalia were noted. She had mild hypotonia of the upper extremities.

A sepsis workup showed elevated inflammatory markers (C-reactive protein (CRP) and procalcitonin). Her basic metabolic panel (BMP) showed normal electrolytes, blood urea nitrogen (BUN) (9 mg/dL; reference range, 8-29 mg/dL), and creatinine (0.50 mg/dL; reference range, 0.8-2 mg/dL). Urinalysis was not performed. Chest X-ray revealed “diffuse interstitial prominence,” consistent with transient tachypnea of the newborn or suspected pneumonia. She passed urine and stool shortly after birth, and her hypotonia resolved a few hours after admission.

She received 48 hours of intravenous (IV) antibiotics (ampicillin and gentamicin), which were discontinued after blood cultures remained negative while she remained afebrile.

At day 3 of life, her respiratory symptoms resolved, and she was weaned to room air. She had an initial postnatal ultrasound at 3 days of life, which was repeated on day 6 (findings described in the next section).

A voiding cystourethrogram (VCUG) was performed on day 6, with findings described in the next section. She maintained adequate urine output throughout her admission, with no signs of urinary outlet obstruction. She was discharged home on day 6 of life on amoxicillin 50 mg daily, as recommended by the pediatric urologist, with a follow-up appointment in the urology clinic, where further diagnostic imaging was pursued. 

Diagnostic imaging

Postnatal Ultrasound (Days 3 and 6)

**Figure 1:**
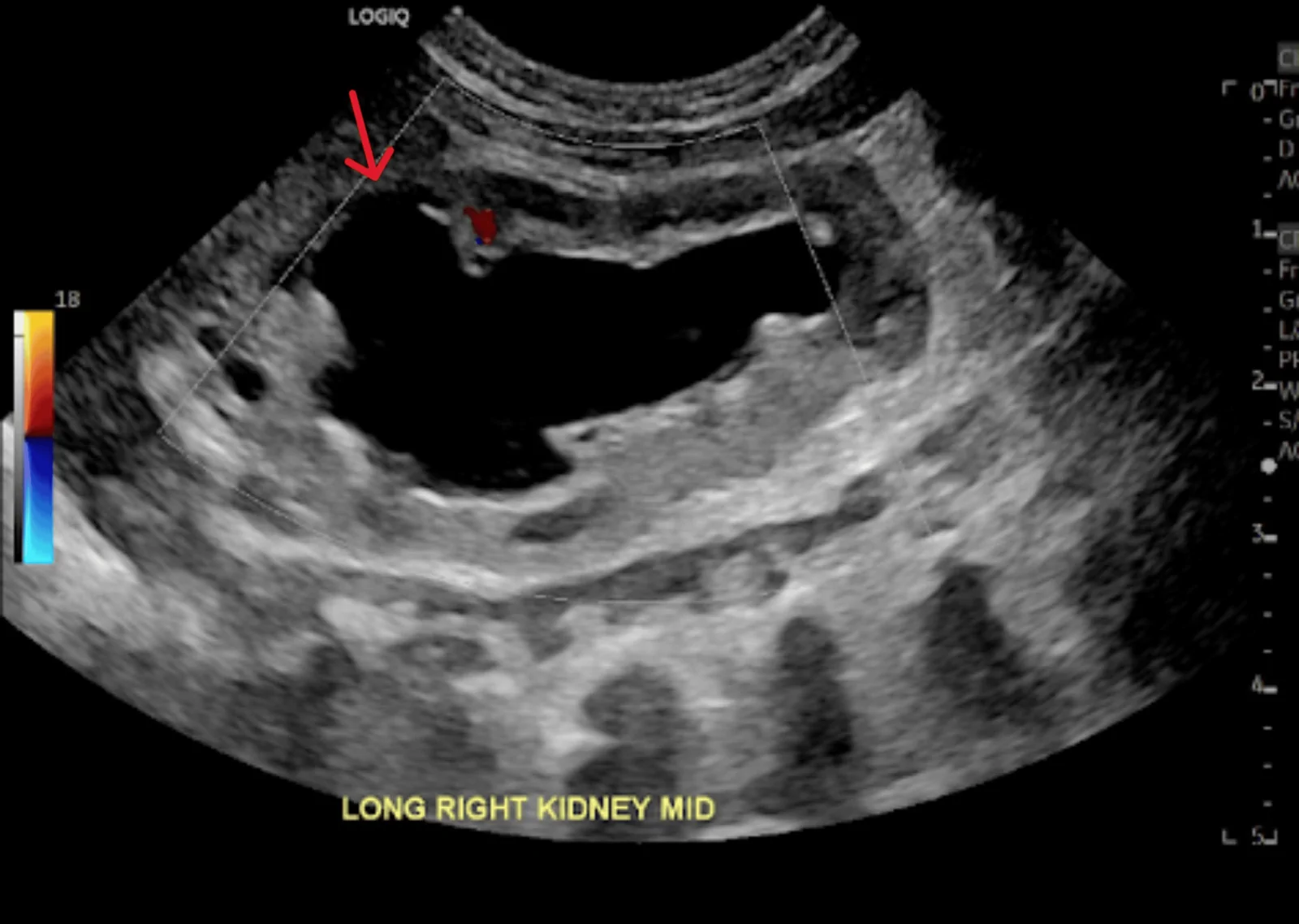
Longitudinal view of the right kidney (5.4 cm) showing grade 4 hydronephrosis, more prominent in the upper pole moiety (red arrow).

**Figure 2:**
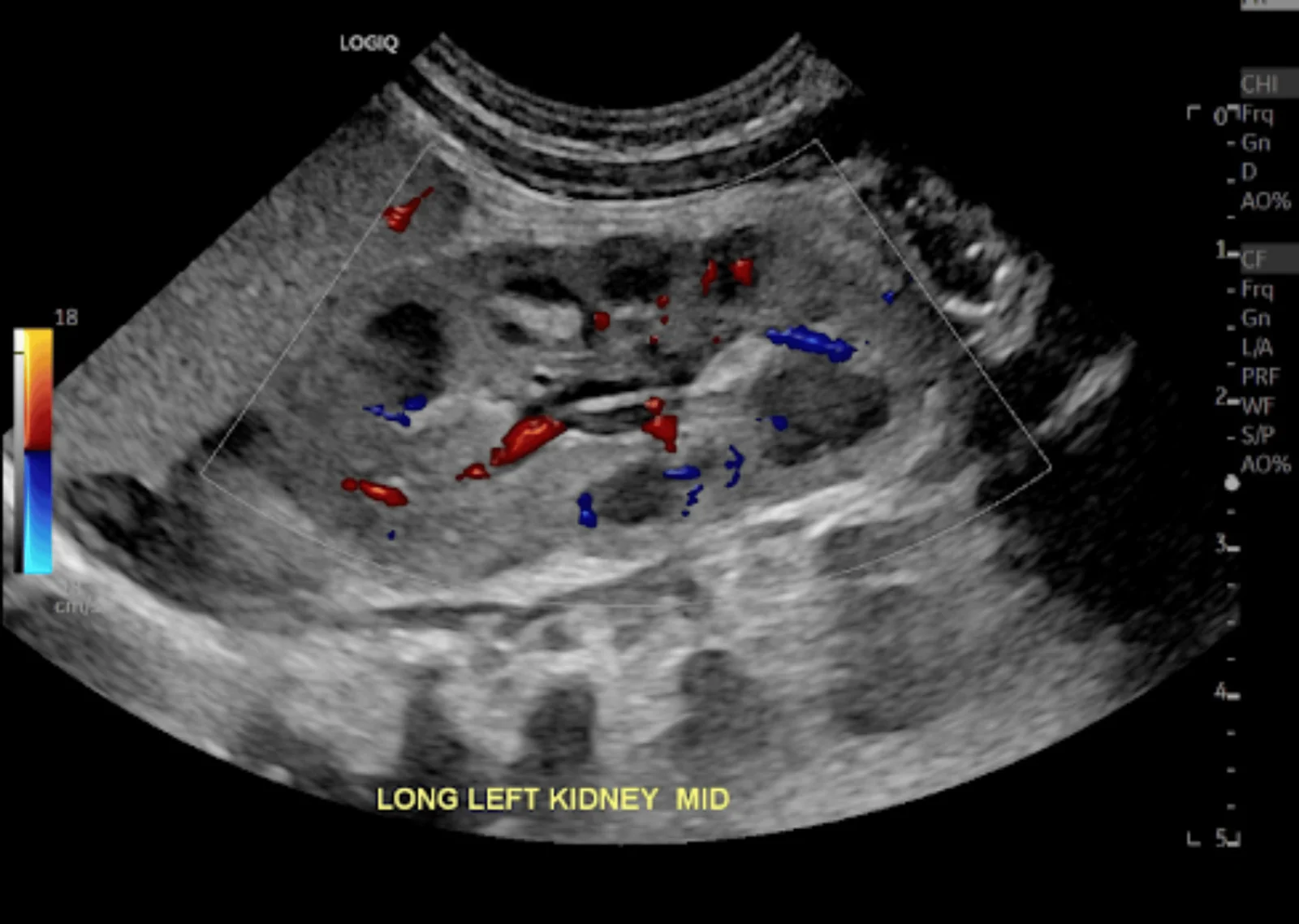
Longitudinal view of the left kidney (4.7 cm) showing normal echotexture with no evidence of hydronephrosis.

**Figure 3:**
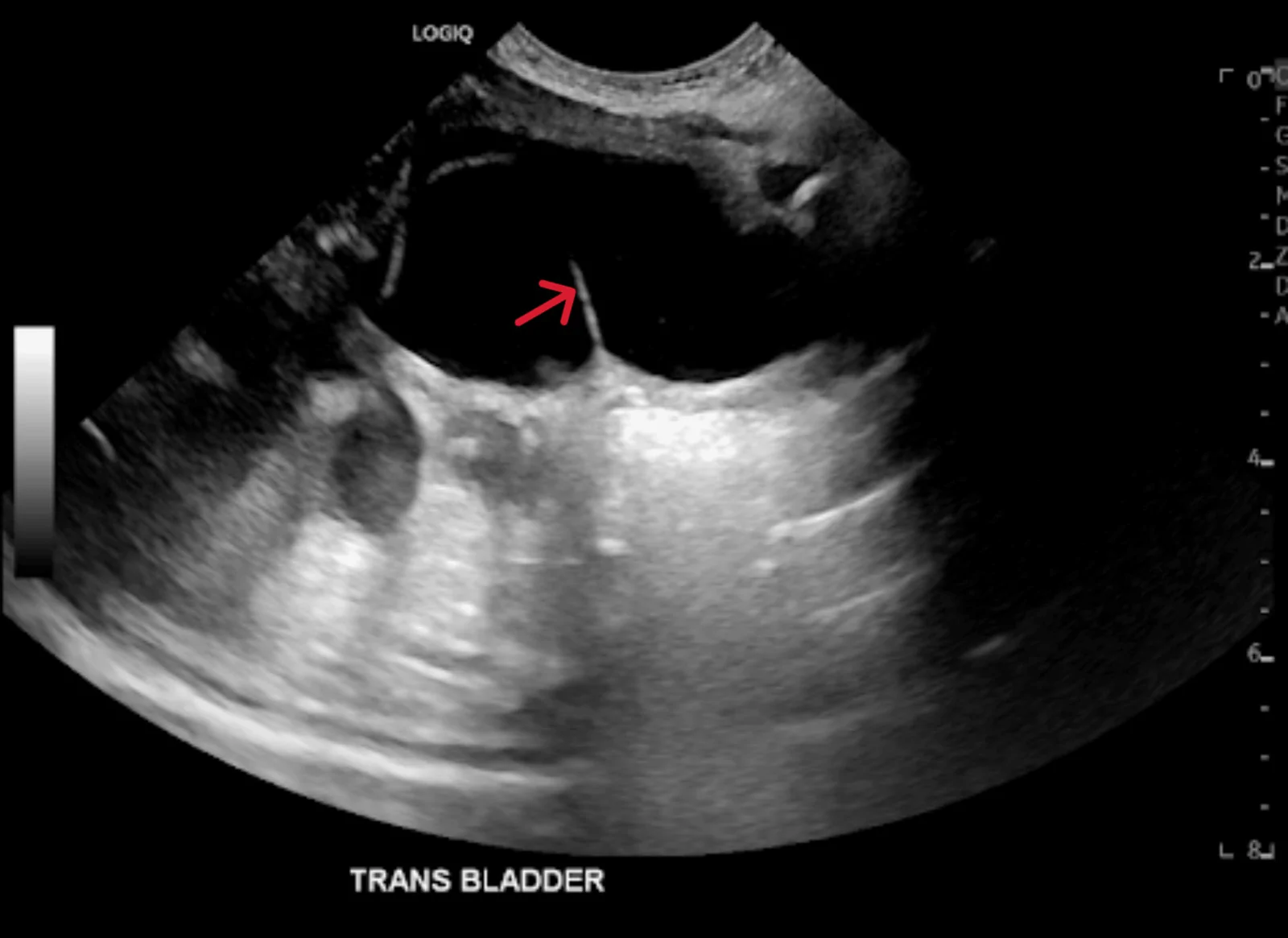
Transverse view of the bladder showing a large intravesical ureterocele (red arrow) at the distal end of a tortuous right ureter.

Confirmed grade 4 hydronephrosis of the right renal pelvis and a right-sided ureterocele (Figure 1). The right kidney was noted to be larger than the left; the left kidney was within normal limits (Figure 2). A transverse image of the bladder showed a large intravesical ureterocele at the distal end of a tortuous ureter (Figure 3).

Voiding Cystourethrogram​ ​​​​​​(Day 6)

**Figure 4:**
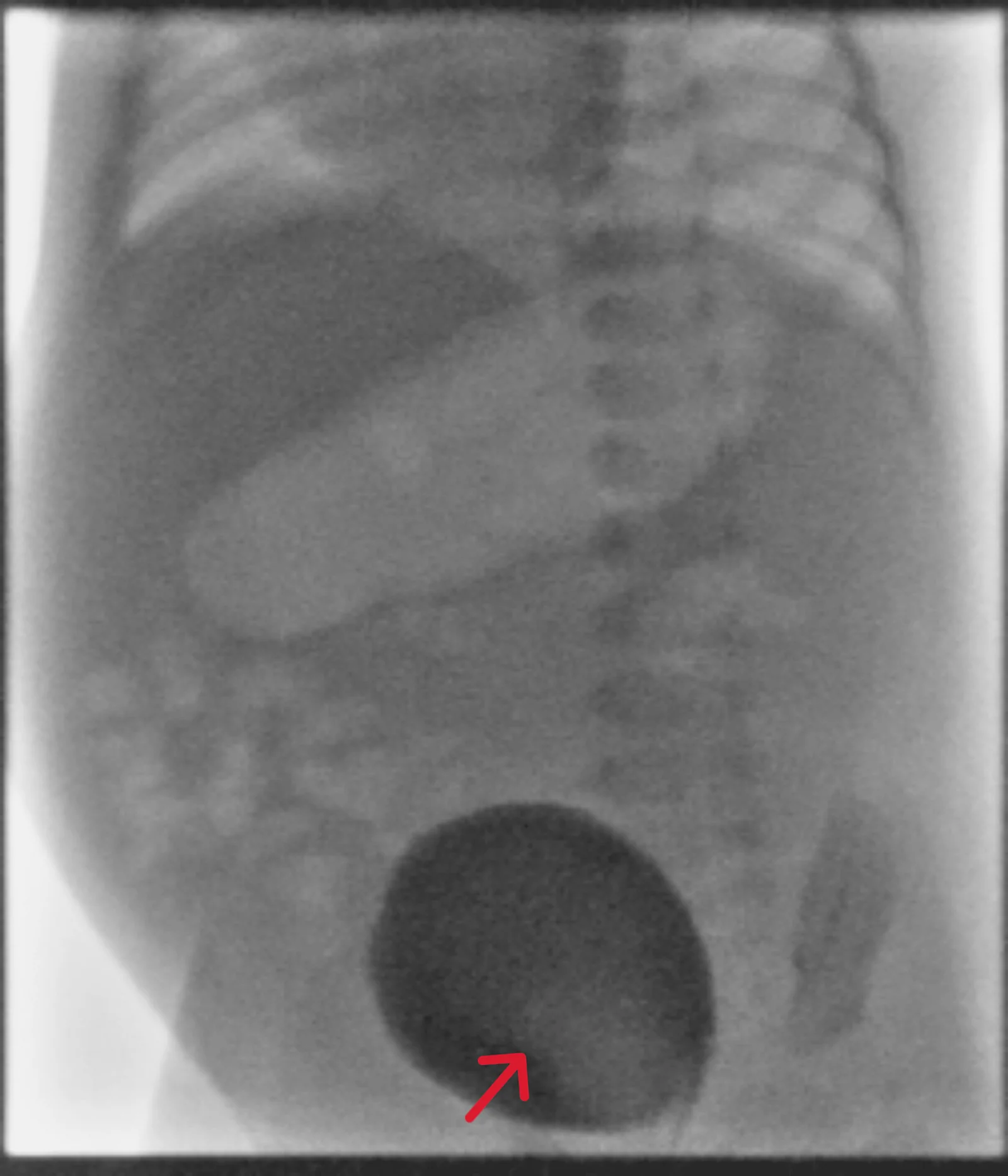
VCUG filling phase demonstrating significant circular filling defect in the bladder lumen (red arrow) corresponding to the ureterocele. VCUG, voiding cystourethrogram

Demonstrated a large, smoothly marginated filling defect at the bladder base indicative of a large right-sided ureterocele (Figure 4). No vesicoureteral reflux (VUR) was detected. However, multiple attempts to initiate voiding by manipulating the catheter were unsuccessful.

Renal Arterial Duplex Ultrasound Scan

At one month of life, it showed a right-sided duplicated collecting system, with the right kidney measuring 6.6 cm compared with the left kidney measuring 5.2 cm. A dysplastic upper moiety with moderate hydronephrosis, increased parenchymal echogenicity, and poor arterial and venous perfusion in the upper pole was noted. A large intravesical ureterocele was identified at the distal end of a tortuous right ureter.

Nuclear Renal Scan With Diuretics

It was performed at three months of life and showed a normally functioning left kidney. The right kidney exhibited a *patulous* pelvis but demonstrated a positive response to diuretics. The upper pole of the right kidney appeared atrophic, with small cystic changes.

Current status and management

The patient remains asymptomatic from a urological standpoint. She was discharged on continuous antibiotic prophylaxis (CAP, amoxicillin) to mitigate the risk of UTIs. She is currently being monitored by pediatric urology for definitive surgical planning (ureteroureterostomy).

## Discussion

A ureterocele is a cystic dilation of the distal portion of the ureter, occurring four to six times more in females compared to males, and the incidence ranges between 1/500 and 1/4,000 [2]. It is usually detected on prenatal ultrasound. It is associated with other congenital anomalies of the kidney, such as a duplex collecting system.

It can be intravesical or extravesical (also known as ectopic) and is usually found in patients with a duplex collecting system.

It can lead to severe hydronephrosis and hydroureter, which can predispose to UTI and renal failure. Other symptoms include obstructive urinary symptoms, abdominal mass/distention, and hematuria [2]. Although our patient did not have any clinical symptoms, she had severe hydronephrosis on the affected side, which could potentially lead to renal failure.

Postnatal evaluation of ureterocele, aside from repeat renal ultrasound, includes VCUG to check for VUR. However, there have been recent concerns regarding radiation exposure and the need for bladder catheterization. Evaluating for VUR is important, as the presence of VUR was found to be associated with female gender, UTI at admission, and recurrent UTI at follow-up (*P* < 0.05) in a study by Şimşek et al. [3]. However, there was no difference between groups with or without VUR in terms of ipsilateral hydronephrosis, scar formation, and the need for surgery (*P* > 0.05) [3]. 

Technetium-99m mercaptoacetyltriglycine (MAG-3) is the gold standard for the evaluation of renal function in patients with ureterocele and is one of the most important imaging modalities during follow-up. Dimercaptosuccinic acid (DMSA) scintigraphy should be undertaken routinely to assess the distribution of function in the duplex kidney and to detect scarred tissue and non-functioning upper poles. In a study by Günşar et al. [4], nearly 55% of patients were found to have renal scarring, which was indicative of parenchymal disruption due to dysplastic changes, UTI, and VUR. 

For management, an individualized approach is recommended. Current academic trends favor minimally invasive surgical techniques or non-operative strategies (a conservative approach for asymptomatic patients with clinically insignificant obstruction and preserved renal function, as upper tract dilation may gradually improve over time).

Indications for surgery include: symptomatic VUR refractory to medical management; a nonfunctioning or poorly functioning moiety causing recurrent UTIs, obstruction, or incontinence; ureterocele causing obstruction, infection, or bladder outlet obstruction; ectopic ureter causing incontinence; and ureteropelvic junction obstruction (UPJO) with progressive hydronephrosis or declining function.

Our patient in this case report qualifies for a surgical approach due to significant obstruction and hydronephrosis caused by the ureterocele, which may eventually lead to a decline in renal function.

Surgical management of duplex collecting systems is individualized based on the clinical presentation, anatomy (upper vs. lower pole involvement, complete vs. incomplete duplication), moiety function, and associated pathology (VUR, ureteropelvic junction obstruction, ureterocele, or ectopic ureter). Table 1 summarizes different surgical approaches.

**Table 1:** Different surgical approaches by pathology, indications, and outcomes.

Surgical options	Pathology	Key points	Outcomes
Upper pole heminephrectomy	Nonfunctioning upper pole moiety	Higher rates of secondary procedures in patients with co-existing vesicoureteral reflux (VUR ) or ureterocele	-Prevents the need for bladder surgery in 97% of patients [5] -Higher risk of secondary procedures in females with high-grade VUR
Ureteroureterostomy/common sheath reimplantation	Nonfunctioning upper pole moiety with VUR	Anastomosis of obstructed upper pole to ipsilateral lower pole, safe alternative to upper pole heminephrectomy	-Lower rates of additional lower urinary tract procedures
Endoscopic transurethral incision (TUI)	Ureterocele	Initial intervention in neonates with sepsis or obstruction, usually a temporizing measure	-56% cure rate in single system -15% cure rate in duplex system [6] -Majority of patients need secondary surgery.
Dismembered pyeloplasty	Ureteropelvic junction obstruction (UPJO)	Preferred for lower UPJO with >3 cm from the ureteral confluence	Favorable surgical success rates [7]
Pyelopyelostomy/pyeloureterostomy	UPJO	Preferred for incomplete duplex systems or when the UPJO is close <3 cm to the ureteral confluence	Excellent outcomes comparable to dismembered pyeloplasty [7]

According to a 2024 meta-analysis by Salehi-Pourmehr et al. [8], patients with a duplex collecting system have a 2.5 times higher risk of requiring secondary surgical intervention compared with those with a single-system ureterocele [8]. While endoscopic techniques, such as transurethral incision (TUI) or holmium laser puncture (the *watering can* technique), provide effective immediate decompression, they are primarily considered temporizing measures. These approaches serve to stabilize the patient and protect renal function while awaiting definitive surgical correction. They are useful in neonates or infants to allow them to grow prior to undergoing more invasive procedures.

To mitigate the long-term risks of de novo VUR associated with temporizing procedures, our patient is likely to benefit from an ipsilateral ureteroureterostomy, which involves anastomosis of the refluxing or obstructed upper pole ureter to the ipsilateral lower pole ureter. The efficacy of this definitive approach is well supported; a study by Eftekharzadeh et al. [9] found that 98% of patients showed resolved or stable dilation at 60 months postoperatively. It allows decompression of the obstructed system without the risks associated with dissection near the renal hilum or the healthy lower pole blood supply. It also avoids the risk of bladder and ureteral complications.

CAP is recommended for children with significant obstructive uropathies (such as ureterocele) until surgical correction is performed, according to the European Association of Urology/European Society for Pediatric Urology (EAU/ESPU) Guidelines (2024/2025) [10]. The American Academy of Pediatrics (AAP) also recommends CAP for children under two years of age with severe urinary tract dilation (UTD) to prevent recurrent infections while awaiting definitive imaging or surgery [11]. A systematic review by Hewitt et al. [12] showed that while CAP can reduce the risk of recurrent febrile UTIs, it does not significantly reduce the rate of new renal scarring or permanent kidney damage. CAP is recommended for ureteral dilation >7 mm, as this finding increases the risk of UTI, which was the case for this patient.

Our patient was started on amoxicillin for prophylaxis and has remained asymptomatic, with stable renal function and no interim febrile UTIs, while awaiting a scheduled ipsilateral ureteroureterostomy.

## Conclusions

The management of large ureteroceles in the neonatal period requires a nuanced balance between preventing infection and preserving renal function. As demonstrated in this case, adherence to current EAU/ESPU guidelines and the use of CAP can effectively manage risks in the short term. While CAP may not eliminate the risk of long-term renal scarring, it serves as a vital safeguard against recurrent febrile UTIs during the critical window before definitive surgery. However, it is impossible to generalize from this case alone. Future surgical planning, whether involving endoscopic decompression or more radical reconstruction, must be tailored to the patient’s specific anatomy and functional status.
